# Between forest and croplands: Nocturnal behavior in wild chimpanzees of Sebitoli, Kibale National Park, Uganda

**DOI:** 10.1371/journal.pone.0268132

**Published:** 2022-05-06

**Authors:** Camille Lacroux, Benjamin Robira, Nicole Kane-Maguire, Nelson Guma, Sabrina Krief

**Affiliations:** 1 UMR 7206 CNRS/MNHN/P7, Eco-anthropologie, Hommes et Environnements, Museum National d’Histoire Naturelle, Musée de l’Homme, Paris, France; 2 Sebitoli Chimpanzee Project, Great Ape Conservation Project, Fort Portal, Uganda; 3 UMR 7179 CNRS/MNHN, Ecologie et Gestion de la Biodiversité, Museum National d’Histoire Naturelle, Paris, France; 4 CEFE, CNRS, Université Montpellier, Université Paul Valéry Montpellier 3, EPHE, IRD, Montpellier, France; 5 Uganda Wildlife Authority, Kibale, Uganda; Universidad Miguel Hernandez de Elche, SPAIN

## Abstract

Some animal species have been presumed to be purely diurnal. Yet, they show flexibility in their activity rhythm, and can occasionally be active at night. Recently, it has been suggested that chimpanzees may rarely engage in nocturnal activities in savannah forests, in contrast to the frequent nocturnal feeding of crops observed at Sebitoli, Kibale National Park, Uganda. Here we thus aimed to explore the factors that might trigger such intense nocturnal activity (e.g. harsher weather conditions during daytime, low wild food availability or higher diurnal foraging risk) in this area. We used camera-traps set over 18 km^2^ operating for 15 months. We report activities and group composition from records obtained either within the forest or at the forest interface with maize fields, the unique crop consumed. Maize is an attractive and accessible food source, although actively guarded by farmers, particularly during daytime. Out of the 19 156 clips collected, 1808 recorded chimpanzees. Of these, night recordings accounted for 3.3% of forest location clips, compared to 41.8% in the maize fields. Most nocturnal clips were obtained after hot days, and most often during maize season for field clips. At night within the forest, chimpanzees were travelling around twilight hours, while when at the border of the fields they were foraging on crops mostly after twilight and in smaller parties. These results suggest that chimpanzees change their activity rhythm to access cultivated resources when human presence and surveillance is lower. This survey provides evidence of behavioral plasticity in chimpanzees in response to neighboring human farming activities, and emphasizes the urgent need to work with local communities to mitigate human-wildlife conflict related to crop-feeding.

## Introduction

The distribution of activities over the day is an important facet of animal behavior. Indeed, time itself is a currency that could be optimized, thereby optimizing an individual’s fitness. However, active time is often limited to a specific period of the day. As such, most species are assumed to be exclusively diurnal or nocturnal. However almost 8.5% of mammalian species do not fit within this usual dichotomous classification, and instead show variations in their activity time between day and night (mammals [1, 2]; primates [3, 4]). Such species, which can be active both during the day and at night, are termed cathemeral [5]. Among primates, only Malagasy strepsirrhines (*Eulemur fulvus* [6]*; Eulemur macaco* [7, 8]) are classified as such. Yet within the broad spectrum of diurnal species, there is some evidence of crepuscular or occasionally nocturnal activity: either through direct visual observations (*Cercopithecus mitis albogularis* [9]; *Chlorocebus pygerythrus* and *Papio anubis* [10]; *Theropithecus gelada* [11]; *Macaca fuscata yakui* [12]; *Pongo pygmaeus* [13]; *Pan troglo*dytes [14, 15]) or acoustic signals (*Procolobus rufomitratus tephrosceles* and *P*. *anubis* [9]; *Macaca sylvanus* [16]; *Gorilla beringei* [17]; *Pan troglodytes* [9, 18–21]). As these night observations are rare and considered opportunistic, despite their potential to provide valuable information, these primate species, especially the suborder Haplorhini, have only been studied during the day until now.

In primate species considered diurnal, occasional nocturnal activity is purported to be a response to environmental pressure and constraints. For instance, active time allocation to night periods has been seen as a means to counteract detrimental effect on individuals’ fitness due to harsh environmental conditions during daytime (e.g. by avoiding higher temperature during the day [22, 23]), due to inter-species resource competition (e.g. by avoiding foraging time overlap with a competitive diurnal species [22]), due to predation risk (e.g. by foraging or moving outside of diurnal predator’s active time [12, 24, 25]) or due to food scarcity or lower availability during day time [26–28]. In addition, climate and weather conditions (e.g. temperature, rainfall, humidity and moonlight) can affect primate sleep duration and quality [29, 30].

Changes in human traditional lifestyle may also have been driven by a response to increased proximity between humans and non-human primates [31; for other taxon, e.g. *Ursus arctos* 32], itself the result of human population growth and expansion of anthropised areas. This is best illustrated with the current crisis in palm oil production: large parts of the Indonesian tropical forest have been logged to allow increased palm cultivation, which has reduced the habitat of orangutans (*Pongo sp*.) and thus affecting their survival [33]. In Africa too, cultivated crops are becoming more widespread, encroaching on forest habitat [34], increasing competition for resources. Crop-feeding may therefore be a by-product of both natural resources becoming less available due to considerable deforestation [35, 36] and the nutritional benefits of cultivated foods. These foods are more palatable and easily accessible for primates [37, 38] through increased proximity and thereby familiar [39–41]. However, during daylight those resources are usually actively protected by farmers, sometimes leading to death or severe injuries for the primates [42]. Thus, primates appear to show some behavioral adaptations, such as crop foraging at specific hours when farmers reduced their vigilance against wildlife incursions (*P*. *troglodytes* [14]; *Macaca maura* [43]). In Kibale National Park, Uganda, primates are thought to be responsible for over 71% of crop damage [44, 45]. Nonetheless, the difficulty of directly detecting these foraging events means that, in many cases, crop damage is sometimes misattributed to other species. In the end, the actual impact of crop foraging by a given species is therefore almost always under or overestimated [46].

To compensate the limited direct assessments of the consequence of crop foraging, one can benefit from new technology, such as camera traps which have become a frequently used tool in ecology to survey wildlife day and night at low cost [47]. This recent development has opened new avenues of research, providing a time saving and easy way for indirect monitoring, revolutionizing our understanding of nocturnal activities. Indeed, camera-traps have helped to provide reliable data on crop-feeding events even at night [14]. In addition, they allow the study of elusive animals [48, 49], to document rare-case occurring patterns (e.g. extraction of honey from underground bee nests by wild chimpanzees (*P*. *troglodytes*) [50–52]), ands to carry-on broad and intensive non-invasive sampling (e.g. in chimpanzees [53, 54]). For terrestrial primates specifically, it is also a mean to study non-habituated groups [55], and minimize zoonoses [56–58] meanwhile limiting poaching exposure by minimizing habituation to human presence [59]. Recently, a study using compiled data obtained via camera trapping at 22 sites in Africa confirmed the existence of nocturnal activity in chimpanzees across savannah and forested sites, at a low but non-null rate (0.14%– 9.58% of the recorded videos according to sites due to different sampling effort and openness of the habitat). Nocturnal activity was more likely at locations with lower levels of human activity, higher average daily temperature the previous day, and/or with a larger percentage of forest composing the habitat [15]. Nocturnal events in the forest were carried out by different individuals, with immatures individuals being the most present, followed by lone adult males [15]. Previously, Krief et al. [14] documented for the first time frequent nocturnal activities of chimpanzees living in a home range bordered by cultivated fields in Sebitoli, in the northern part of Kibale National Park in Uganda. Such results contrast with Tagg et al.’s study [15]. In Sebitoli, chimpanzee nocturnal activity appears paradoxically linked to human presence (i.e farming activity). In addition, active chimpanzees were male and female of all ages. However, Krief et al’s study period was short and the survey focused on a single maize plantation.

To clarify this preliminary result, we conducted a follow-up study on an extended time period in Sebitoli. We monitored the nocturnal activity of this chimpanzee community over a larger part of their home range using up to 16 camera-trap set-ups over 18 km^2^ during 15 months. Camera-traps were located both inside and at the forest edge, in order to compare data from other sites and to explore the influence of anthropisation of the forest edge [15]. In the case where frequent nocturnal activities of chimpanzees have been confirmed inside and at edge of the forest, we aimed to understand whether environmental conditions might have driven such a change in lifestyle in both locations, and which individuals are more likely to be involved. We therefore formulated the following (but not mutually exclusive) hypotheses, following previous studies [14, 15]:

### 1) Harsh meteorological conditions reduction strategy

Nocturnal activities may be more likely when harsh conditions (high temperature or heavy rains) occurred during the previous day, having reduced the opportunity to practice any activity during the day. We hypothesized that chimpanzees may delay their activities until evening or night in order to face tempered conditions, such as cooler temperatures, and thus avoid physiological stress.

### 2) Human avoidance strategy

i. Nocturnal activities may be a result of risk associated with and thus avoidance of humans. In Sebitoli, the main life-or-death risk faced by immature and adult chimpanzees is humans (farmers and poachers). Farmers are working in their field and actively guarding them during the day, chasing wildlife detected in, or near the fields, sometimes with spears. However, they usually do not patrol more than once or twice during the night when they are mainly vigilant for elephant (*Loxodonta sp*.) incursion. As such, we predicted that the categories of chimpanzees engaging in nocturnal behaviors will differ in activity and according to the location. Adult males may engage more in dangerous nocturnal activities in crop fields. On the other hand, we expected adult females with infants and juveniles to be less engaged in nocturnal activities in the fields, likewise for other vulnerable individuals such as those with physical ailments/mutilations. Furthermore, group size could be smaller when entering the gardens as a strategy to be less easily spotted by humans [46].

ii. Light levels might also be a factor shaping nocturnal activity rate. Preliminary results of the first study relating to this community seemed to indicate that chimpanzees could favor moonless nights, potentially to decrease the risk of being seen by farmers [14] and in accordance with lunar phobia seen in other cathemeral mammals [3]. Indeed, human vision is more suited for light condition, and farmers or guards in Sebitoli are not affluent (Krief, personal communication) and do not often have the financial means to be equipped with light torches. Such a hypothesis might be valid in a crop context where the habitat is open, whereas one can consider moon illumination as a facilitating condition for navigation would hold particularly in a dense habitat such as forest context, where visual abilities are already greatly impeded [60]. Thus, we predicted that the impact of moonlight would differ depending on chimpanzees location, with higher nocturnal activity in forest during bright night and higher nocturnal activity in fields during dim and waning moonlight.

### 3) Complement dietary requirements strategy

i. Tropical forests have traditionally been considered permanently food-rich environments [61]. Botanical investigations however revealed a more nuanced picture: for most species, trees fruit in bursts, either sub-annually or pluri-annually [62, 63] creating seasonal and inter-annual fluctuations in fruit quantity. As such, food quantity alternates from scarce to plentiful for frugivorous dominant species, such as chimpanzees in Kibale [64]. In Kibale forest (Kanyawara and Ngogo research sites) for instance, fruit availability reaches a maximum after the first wet season ends, while it is at its lowest in the preceding months, with fruiting tree density about six times lower than during peak fruiting [62]. Such seasonal low availability of wild sources of energy food in the forest may result in night feeding of highly energetic resources, as chimpanzees fail to meet their food requirement during the day. Hence, we predicted that low wild fruit availability would trigger increased night activities in both forest and crop fields.

ii. The presence of a highly nutritious food items represented by maize crops could be a strong motivation for chimpanzees to forage at night. We predicted that the presence of maize would increase nocturnal activity within the forest, as chimpanzees will travel to reach the fields if they nested far from the park edge. In parallel, we predicted that nocturnal activity in the fields would increase during the maize season.

To test these predictions, we analyzed the 1 808 clips containing chimpanzees recorded over the time period. We described chimpanzee behavior, party composition, and timing of nocturnal activity. Next, we tested correlative patterns between the probability of nocturnal activity and a set of environmental predictors (temperature, rainfall, moon illumination and food availability) using a linear modelling approach [65] separately for each habitat type (forest or cultivated fields). Overall, by providing a detailed picture of nocturnal behaviors in chimpanzees, we finally hope to contribute to a better understanding of changes to chimpanzee ecology arising from habitat modification.

## Material and methods

### Field site

**Fig 1:**
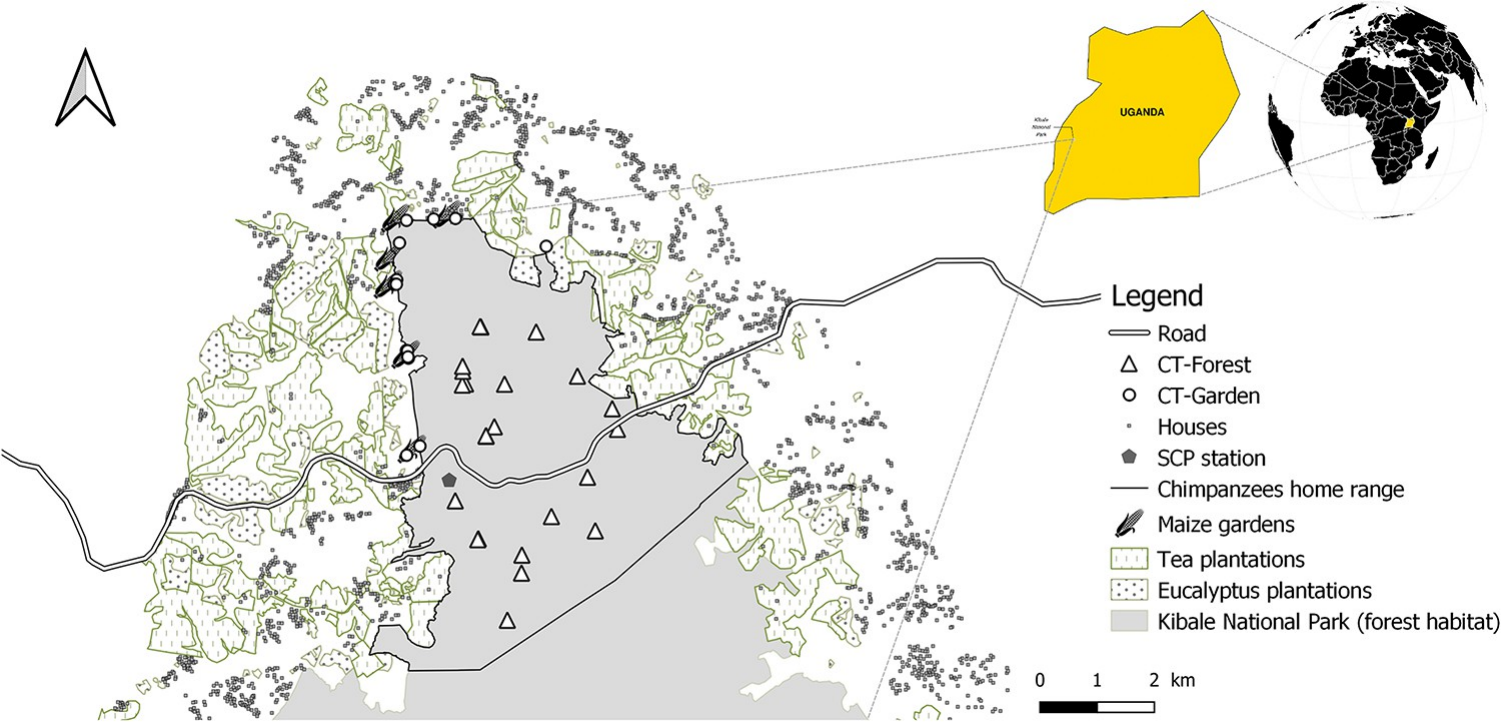
Map of Sebitoli chimpanzee forest home range and surrounding cultivated fields, Kibale National Park (KNP), Uganda. Republished from [73] under a CC BY license, with permission from Revue Francophone de Primatologie, original copyright 2012.

Sebitoli chimpanzees home range is located in the North part of Kibale National Park (KNP), Western Uganda (KNP: 795 km^2^, 0°13’-0°41’N and 30°19’-30°32’E [66]; Fig 1). The park is a medium-altitude moist tropical forest that is described as a biodiversity hotspot [67]. The climate of this equatorial zone is typical of rainforest areas, with two rainy and two dry seasons [62]. In the Northern part of the park, the forest border is surrounded by numerous agricultural plots such as small-scale farms with food crops (72%), tea (24%) and eucalyptus plantations (4%) [68]. The forest itself is composed of regenerating forest (35%), degraded forest (35%), followed by mature forest (14%), terrestrial herbaceous vegetation (14%) and patchy shrub/wetland vegetation (1%) [69]. A tarmac road with heavy traffic divides the community’s home-range into two parts: “Sebitoli North” and “Sebitoli South” [70, 71]. A trail system consisting of 120 km of transects directed north-south and east-west was created to facilitate movements of our teams. Chimpanzees also frequently use these trails.

### Chimpanzee community

Since 2009, the Sebitoli Chimpanzee Project (SCP) team has been monitoring the chimpanzee community of the Eastern subspecies (*P*. *troglodytes schweinfurthii*) on a daily basis, which numbers around 80 individuals, 66 of which have been identified by habituation. Nevertheless, some individuals are still in the process of habituation. Crop fields are only present in the western and northern parts of Sebitoli North (Fig 1). In “Sebitoli South” (East and West) and in the eastern part of “Sebitoli North”, the fields bordering the forest were mainly composed of tea gardens at the time of the study, which are not consumed by chimpanzees. Sebitoli chimpanzees exhibited a high level of limb anomalies almost certainly caused by wire snares [71]. Using camera traps, we recognised 62 individuals (94% of identified individuals) including 13 adult males (over 15 years), 23 adult females (over 15 years), 17 immature individuals (juveniles from 3 to 9.9 years and sub-adults from 10 to 14.9 years) and 9 infants (under 3 years and dependent of their mother) [72]. In the following analyses, the 9 females with clinging infants and the 6 mutilated individuals (2 adult males, 2 adult females and 2 immatures) were classified as “vulnerable” (less likely to respond as fast to a danger because of the infant or the injured leg or arm) and thus excluded from their age-sex categories.

### Data collection

#### Camera trap and chimpanzee behavior

Camera traps (CT) were set from the 3^rd^ January 2017 to the 6^th^ April 2018 within the home range of Sebitoli chimpanzees (forest) and at its border with cultivated fields of maize (henceforth fields). Camera traps consisted of HD Reconyx XR-6 Ultrafire™ (30s-long, 4s delay, sensed up to 21m) and Bushnell Trophy Cam HD Max™ (60s-long, 5s delay, sensed up to 18m) with a day/night auto sensor and sound recordings. The cameras were not placed randomly but with the objective of maximizing the probability of capturing images of chimpanzees from head to toe in order to identify them. Any information such as a missing ear, finger or toe can help in identification. The cameras were therefore placed 1 m above the ground and at least 1 m from an open area where our field teams, who followed them on a daily basis, estimated that they would pass. In the forest, CTs were set up on junctions of open trails frequently used according to long term data regarding the core area. At the edge between the forest and the maize fields, CTs were set up when chimpanzees were reported by farmers or by our team, who on a weekly basis surveyed for footprints, food remains (eaten corns as well as wadges of stems) and/or feces. They were positioned in different fields, so we assumed the clips to be independent as they do not show the same crop visiting event. Due to logistical challenges and technical issues (e.g. camera traps damaged by elephants or poachers; memory reaching full capacity), the entire study-period was not covered for some camera traps. In the forest, 14 378 relevant clips, obtained by on average 9 camera traps per day (min = 3, max = 13), spanning 448 days with functional camera traps, that is 4002 trap days, were analyzed. In the maize fields, 4 778 relevant clips obtained by on average 3 camera traps per day (min = 1, max = 4), spanning 197 days with functional camera traps, that is 607 trap days, were analyzed. Among them, only a part was triggered by chimpanzee presence (see Results).

For each clip, we identify all animal species observed, the respective number of individuals, sex, age class, identity whenever possible. For all the individuals, we also recorded all occurrences of the following behaviors: travelling, sitting, standing, resting, grooming, feeding on wild food and cropfeeding. We summarized it in ethogram available in (S1 File). The longest activity of an individual or the most frequent activity among several individuals was considered to determine the main activity for a given clip.

#### Food availability

To estimate wild food availability, we used monthly phenological surveys collected during the study period. These included 373 trees of 39 species along 10 trails of 500 m located within the chimpanzee home range. We calculated a food availability index (FAI) by quantifying fruit abundance per month, using a ‘percent basal area fruiting/ha’ method [see 69]. Finally, to determine the presence of maize, a team patrolled at the edge of the chimpanzees’ home range and made weekly estimates of the maize maturity.

#### Data analysis

**Fig 2:**
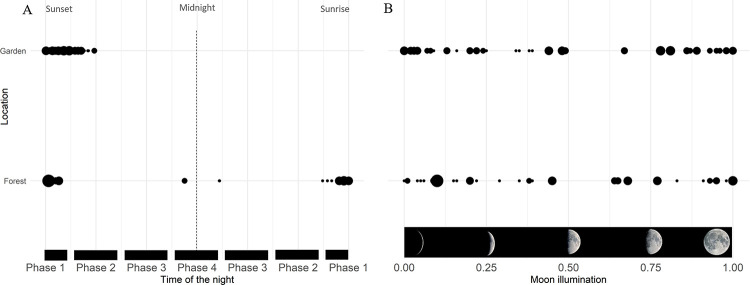
Number of chimpanzees observed with camera traps located nearby fields and within the forest of the Kibale National Park, Uganda in Sebitoli area according to the phase of the night (A) or moon illumination (B). A: To be comparable between fields and forest and times of year, the recording time of the clips was scaled from 0 to 1 based on the time difference from sunset on that day (or the previous day for clips captured after midnight) and night duration (Time of the night = (Tvideo—Tsunset)/night duration). Each dot represents a video with sized by the maximum number of individuals observed, respectively for field or forest habitats. The vertical gray line indicates approximately an hour. Night time was separated into four phases represented by the horizontal black bars at the bottom: phase 1 (1 h after/before sunset/sunrise), phase 2 (within 2 h of twilight); phase 3 (2 h of early morning and late night), phase 4 (2 h around midnight represented by a dotted line). B: Each dot represents a video with and is sized by the maximum number of individuals observed, respectively for field or forest locations. The vertical gray lines indicate the moon illumination, while the bottom illustration depicts their equivalence in “moon phase”.

Two viewings of each clip (one by SCP field assistant dedicated to CT project and one by CL) were performed (S2 File). Clips were considered non-independent if they were recorded less than 15 minutes apart: they formed one “event” [15]. We estimated the party size by compiling the number of different identified individuals seen across all clips of a same event. Then, we obtained the number of chimpanzee occurrences by adding the party size estimated for each event. To each event, we associated to the given date the following descriptive variables: 1) time of *sunset*, *sunrise* and civil *twilights* -defined as the period before the sun reaches 6° under horizon when human eye clearly distinguishes terrestrial objects- (using the web tool https://www.timeanddate.com/sun/). We defined true night as the period of time occurred between sunset and sunrise, it was split into 4 phases (see legend Fig 2, following [15] classification), 2) the *moon illumination* (the measure of the incident moon light illuminating a surface (using the U.S Navy web tool http://aa.usno.navy.mil/data/index.php) 3) the *FAI* per day which was linearly extrapolated from the monthly value 4) the presence of *maize* crops in the fields 5) the mean *temperature* from the previous day (Chapman, personal communication) 6) the *rainfall* from the previous day in Kibale National Park (Chapman, personal communication) (S3 File).

### Statistical analyses

In order to investigate whether environmental, risk avoidance or food constraints influenced chimpanzee nocturnal behavior (hypotheses 1, 2 and 3 respectively, see Introduction), we analyzed the joint effect of variables describing these constraints on the probability of observing nocturnal activity in the forest and in the fields separately (over 448 nights for the forest model and 197 nights for the garden model). As such, we used a generalized linear model approach (GLM) [74, 75], with binomial structure and a logit link error function [75] since the output variable was whether we observed nocturnal behavior on a given night. To control for sampling effort, we integrated the number of camera trap active at the observation data as an offset variable in the model. In order to test for the harsh conditions reduction strategy hypothesis (hypothesis 1), we included as explanatory variables, rainfall intensity (total mm of rain) and mean daytime temperature (in Celsius) of the day preceding the night as tested predictors. In order to test for the human avoidance strategy hypothesis (hypothesis 2), we included the moon illumination of the night of interest as a supplementary tested predictor since this variable might represent perceptibility. In order to test the food-complementation strategy hypothesis (hypothesis 3), we included the FAI per day for the period corresponding to the night of interest, as a tested predictor. For the presence of maize (binary variable, “yes” or “no”), it was only integrated into the forest model, as chimpanzee’ visits to the fields only happened when maize crops were present. Furthermore, we considered that foraging strategy in a given environment depends simultaneously on food availability and human risk. Hence, we put initially the variables moon illumination and FAI in interaction. Similarly, as we expected raid frequency to be related to environmental conditions, e.g by increasing concurrent with severity of conditions, we initially interacted maize presence with temperature and rainfall for the forest model.

To allow comparison among estimates, all continuous variables were scaled to a mean of 0 and a standard deviation of 1 (i.e. z-transformation [76]). Previously, when data were skewed, we applied transformations in order to reach distributions that are more symmetrical. This was notably true for rainfall that was log transformed. However, the variable remained right-queued. As removing extreme (but still reliable) high rainfall values did not substantially change the output, we present results from the entire data. We confirmed there was no major internal correlation issues based on the Variance Inflation Factor (VIF) (max VIF < 2.06 in the forest model and < 1.13 in the field model [77]), using the “vif” function from the “*car*” package [78].

While all models initially included all the above-mentioned interactions, we presented the latest version of the models with only the significant interactions.

We assessed the joint effect of variables by comparing the *full* model, including the tested predictors as well as the control variables, to the null model, including only control variables or a constant if none [79], using a likelihood-ratio test (R-function “anova” set to “Chisq” [75]). In case this comparison was significant, we then tested for the singular effect of each variable by comparing the *full* model deviance and the deviance of a *reduced* model [79] excluding the variable of interest one at a time, using “drop1” function [80]. In order to account for multiple testing downside, we maintained the false discovery rate to the nominal value of 0.05 using Benjamini and Liu’s second procedure [81]. For each model analysis separately, that is considering p-values given by the “drop1” function and the likelihood ratio test between the *full* and *null* model, non-significant interactions were discarded once. We qualify a variable as significant only if it occurred below the corrected threshold following this last procedure.

We visually checked the models’ assumption (e.g. homogeneous distribution of residuals vs fitted values), and assessed the models stability using various parameters such as leverage values (forest model: 0.031; fields model: 0.061), the maximum Cook’s distance (forest model: 0.079; garden model: 0.088), the maximum Dffits (forest model: 0.569; fields model: 0.801) and the maximum DFBetas (forest model: 0.102; fields model: 0.168). It indicated no obviously influential cases [82] (S4 File). Furthermore, overdispersion was not an issue in the forest model (overdispersion test, parameter = 1.832, χ^2^ = 809.715, df = 442, *p*-value<0.001) or the fields model (overdispersion test, parameter = 1.439, χ^2^ = 276.236, df = 192, *p*-value<0.001).

All statistical analyses were performed in R software, version 3.3.2 [83].

### Ethical guidelines

This research was conducted in the context of the Memorandum of Understanding MNHN/UWA/Makerere University SJ 445–12 following the guidelines of the Uganda Wildlife Authority (UWA).

## Results

### Active time distribution

Out of the 19 156 relevant clips recorded during the study period, 1 808 (9.4%) clips involved chimpanzees were pooled in 939 events. In the forest, out of the 796 events involving chimpanzees, 36 (4.6%) happened during night time. In parallel, at the edge of the fields, out of 143 chimpanzees events, 57 (39.9%) were recorded at night (Table 1).

**Table 1:** Number of clips and events recorded in the forest and at the edge of the gardens during day and night. A total of 14 camera-traps were used over 2828 days from 03 January 2017 to 06 April 2018 in Sebitoli area, Kibale National Park, Uganda.

	Camera-trap days ^a^	Number of clips ^b^	Number of clips including chimpanzees	Number of chimpanzees clips during nighttime	Number of events ^c^ with chimpanzees			
					Total	Day ^d^	Twilight ^e^	True Night ^f^
Forest ^g^	2 210	14 378	1 353 (9.4%)	44 (3.3%)	796	760 (95.5%)	26 (3.3%)	10 (1.3%)
Fields ^h^	618	4 778	455 (9.5%)	190 (41.8%)	143	86 (60.1%)	18 (12.6%)	39 (27.3%)

^a^ Sampling effort as the number of camera traps active during the recorded period.

^b^ Videos recorded because of the presence of an animal filtered out non-desired records due to wind in vegetation for instance.

^c^ A clip or a set of clips captured in which chimpanzees are seen in less than 15min apart from the next one with chimpanzees.

^d^ Period between sunrise and sunset.

^e^ Period 30 min before sunrise to sunrise and sunset to 30min after sunset.

^f^ Period between twilights.

^g^ Camera traps inside the Forest.

^h^ Camera traps on the edge of the forest near a maize field.

At the forest-fields edge, nocturnal activity occurred mainly in the early hours of the true night, especially in the phase 2 of the night (i.e. between one and two hours after sunset). No event occurred after 22h and no chimpanzees were detected in the early morning at the edge of the fields (Fig 2A). Inside the forest, nocturnal activities recorded were more frequent in twilight than in true night (26 vs 10 out of 36 events) (Table 1). Most nocturnal activity occurred in the early morning, within an hour before sunrise. In addition, some nocturnal activities occurred during the deep phase of the night (at 00h38 and at 01h40) (Fig 2A).

### Activity allocation during night time

Locomotion and foraging activities were the most frequent behavior recorded at night. 144 out of 284 (50.7%) recorded behavior occurrences corresponded to travelling and 129 (45.4%) corresponded to foraging- (Table 2). Nocturnal activities were significantly different according to the habitat (χ^2^ = 135.280, df = 3, *p*-value<0.001) with “travelling” as the most frequent activity in the forest. 93 out of 99 (93.3%) nocturnal activities were travelling occurrences in the forest and 51 out of 185 (27.6%) in the garden. No feeding activities occurred in the forest while cropfeeding represented129 (69.7%) of behavior occurrences in the garden). In fact, foraging behaviors on wild food was not observed at night in the forest. Foraging behaviors were only observed near fields and were related to maize consumption.

**Table 2:** Frequency of occurrence of chimpanzee nocturnal activity in function age-class and sex.

**Behavior**	**Number of individuals implicated in nocturnal behavior**					
	Adult ^a^ male	Adult female with a clinging infant ^b^	Adult female without clinging infant	Immature ^c^	Unidentifiable^d^	Mutilated^e^
**Community composition ^f^**	11	9*2	12	15		6
**Forest**	35	12*2	3	21	10	6
Feeding on wild food	0	0	0	0	0	0
Travelling	33	11*2	3	20	10	5
Sitting/standing/resting	1	0	0	0	0	0
Grooming	1	1*2	0	1	0	1
**Fields**	54	13*2	5	45	21	34
Cropfeeding	34	9*2	5	30	16	26
Travelling	19	4*2	0	13	4	7
Sitting/standing/resting	1	0	0	2	1	1
Grooming	0	0	0	0	0	0

^a^ Non-mutilated individual of over 15 years [84].

^b^ Mother and infant (under 3 years old) are considered together in this category.

^c^ Summation of healthy juveniles (from 3 to 9.9 years old) and sub-adults (from 10 to 14.9 years old) [84].

^d^ Adult chimpanzees for which the sex could not be identified or individuals not identifiable by age.

^e^ Mutilated individuals of all ages and classes excluded from the other categories.

^f^ The community composition was obtained independently of the CT, from direct cumulative observations.

### Group size and composition during night events

**Fig 3:**
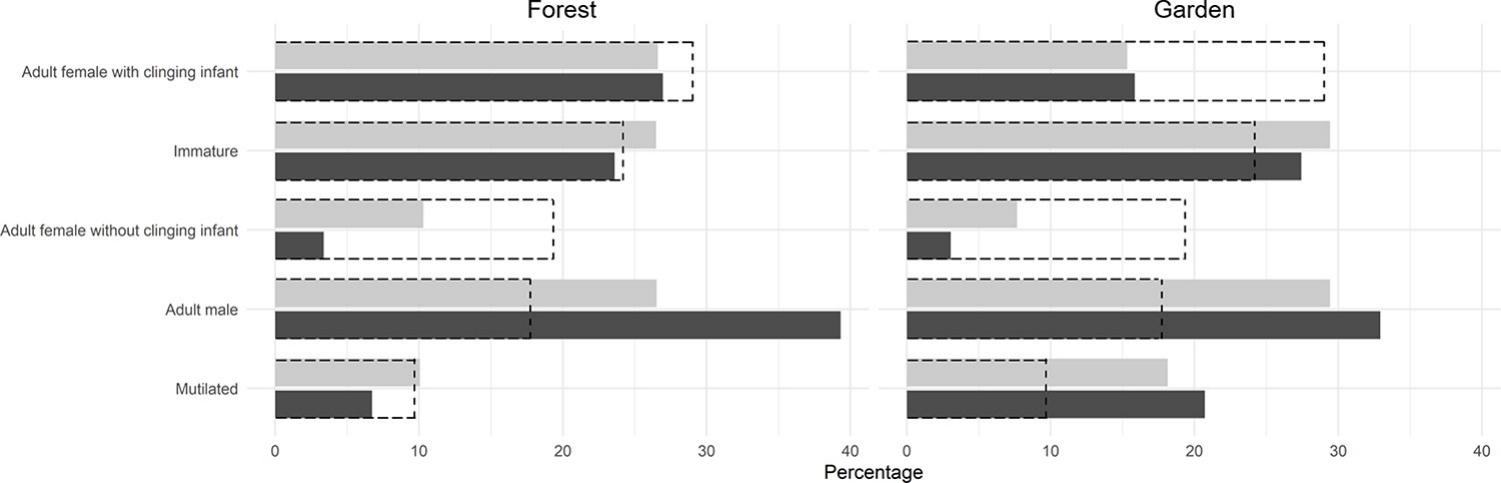
Ratio of occurrences during nocturnal activity of chimpanzees in function age-class, sex and mutilations according to the daytime and the habitat (the individuals not identified have been excluded). Bars represent the percentage of occurrences of age-sex classes during day (light gray) and night (dark gray) time. The dotted bars depict the percentage of an age-sex class in the Sebitoli chimpanzees’ community. Adults are non-mutilated individuals of over 15 years [84]; infants (under 3 years old) are dependent of their mother and counted together; immatures are healthy juveniles (from 3 to 9.9 years old) and sub-adults (from 10 to 14.9 years old) [84]; mutilated individuals of all age and class excluded from the other categories.

Party size observed within the forest or at the forest-field interface did not differ between night and day, whether or not we removed those involving only one individual (all t-test had a *p*-value > 0.05, Table 3). However, when distinguishing habitats, there were more lone individuals at night in the forest (chi-square test, χ^2^ = 10.872, df = 3, *p*-value = 0.012, Table 3). Despite this, events in the fields (day and night combined) included significantly fewer chimpanzees on average than in the forest (t-test, with lone individuals: t = 1.737, df = 208.75, *p*-value = 0.083; without lone individuals: t = 2.094, df = 140.53, *p*-value = 0.038), which suggests considerably lower party-size in the field on average. To determine if a given category, and more particularly vulnerable individuals such as mutilated chimpanzees or females with dependent offspring and immatures, were recorded more often on CT during night event in the gardens to prevent risks of being detected we determined the rate at which each chimpanzee category (see Methods) was seen (Fig 3) and compared it to the community composition (z-test with the Benjamini’s False Decovery Rate (FDR) second correction [82], the significance is therefore indicated relative to the new threshold of 0.002 to maintain the FDR at the nominal value of 0.05). On all camera clips, adult males were the most highly encountered category of individuals, especially at night in the forest compared to their proportion in the community (z-test, χ^2^ = 61.174, df = 1, *p*-value<0.001). Immature individuals of both sexes, adult females with clinging infants and mutilated individuals were equivalently seen day and night or in both habitats compared to their proportion in the community (z-tests, all *p*-value>0.002). Interestingly, adult females without clinging infants were less represented in clips compared to their distribution in the community (z-tests, all *p*-value<0.002) with the exception of clips in the forest during daytime (z-test, χ^2^ = 5.265, df = 1, *p*-value = 0.022).

**Table 3:** Party size and mean group size in function of time condition (day or night) and habitat.

	Event with lone individuals (%)	Mean party size including lone individuals ± sd*	Mean party size (excluding events with lone individuals) ± sd*
Forest during daytime	32.11	3.73 ± 3.61	5.03 ± 3.75
Forest during nighttime	58.33	2.56 ± 3.81	4.73 ± 2.93
Fields during daytime	36.05	3.01 ± 3.19	4.15 ± 3.25
Fields during nighttime	33.33	3.25 ± 2.78	4.37 ± 4.03

* Standard deviation

### Environmental factors influencing nocturnal activity

Here we tested using a generalized linear model whether temperature, rainfall, moon illumination and/or food availability (wild and domestic) influenced the probability of capturing chimpanzee nighttime activity, according to the habitat type (forest or fields) and taking into account the sampling effort represented by the number of active camera traps.

The full model differed significantly from the null model in the forest habitat (χ^2^ = 16.024, df = 5, *p*-value = 0.007) and at the interface between forest and fields (χ^2^ = 13.159, df = 4, *p*-value = 0.011). All initially included interactions, whether triple or pairwise, did not have a significant impact on the model response and were therefore removed (Table 4).

**Table 4:** Results of the GLM on the presence of chimpanzees at night within the forest (model 1) or then the fields surrounding the national park (model 2).

Term	Estimate	Standard deviation (sd)	Lower confidence limit	Higher confidence limit	Degree of freedom (df)	Statistics value (χ^2^)	*p*-value
**Forest model (n = 448 nights)**	PresenceForest ~ Temperature.z + Rainfall.log.z + Moon.illumination.z + FAI.z + Maize, offset = NumberCT.z						
Intercept	-3.083	0.285	-3.681	-2.558			
Temperature.z	0.464	0.227	0.020	0.910	1	4.189	0.041 *
Rainfall.log.z	-0.340	0.229	-0.821	0.087	1	2.391	0.122
Moon.illumination.z	-0.046	0.181	-0.407	0.309	1	0.066	0.798
FAI.z	0.098	0.287	-0.477	0.651	1	0.116	0.734
Maize	0.581	0.524	-0.440	1.619	1	1.238	0.266
**Fields model (n = 197 nights)**	PresenceFields ~ Temperature.z + Rainfall.log.z + Moon.illumination.z + FAI.z, offset = NumberCT.z						
Intercept	-2.220	0.244	-2.736	-1.770			
Temperature.z	0.674	0.235	0.225	1.150	1	8.769	0.003 **
Rainfall.log.z	-0.189	0.284	-0.823	0.320	1	0.478	0.489
Moon.illumination.z	-0.077	0.234	-0.544	0.379	1	0.109	0.742
FAI.z	0.253	0.257	-0.281	0.734	1	0.915	0.339

^a^ Not shown because having a limited interpretation. “.z” predictors were z-transformed to a mean of zero and a standard deviation of one. “.log” predicators were log transform. Presence: presence (1) or absence (0) of chimpanzees on all cameras traps per night according to their location (forest vs garden); Moon.illumination: percent of illumination according to the moon phase (0 no moon night to 1 full moon night); Rainfall: the amount of precipitation measured in mm from the day before; Temperature: mean temperature measured in °C from the day before; FAI: index of wild food availability in the forest; maize: presence (1) or absence (0) of maize in the nearby fields.

Forest model predicators before z-transfromation (mean±sd): Temperature (20.626±1.377), RainfallLog (0.893±1.170), Moon.illumination (0.509±0.351), FAI (1.283±0.483), Number.CT (8.933±2.100)

Garden model predicators before z-transfromation (mean±sd): Temperature (20.809±1.204), RainfallLog (0.585±0.982), Moon.illumination (0.496±0.349), FAI (0.949±0.265), Number.CT (3.081±1.120)

#### Harsh meteorological conditions hypothesis

**Fig 4:**
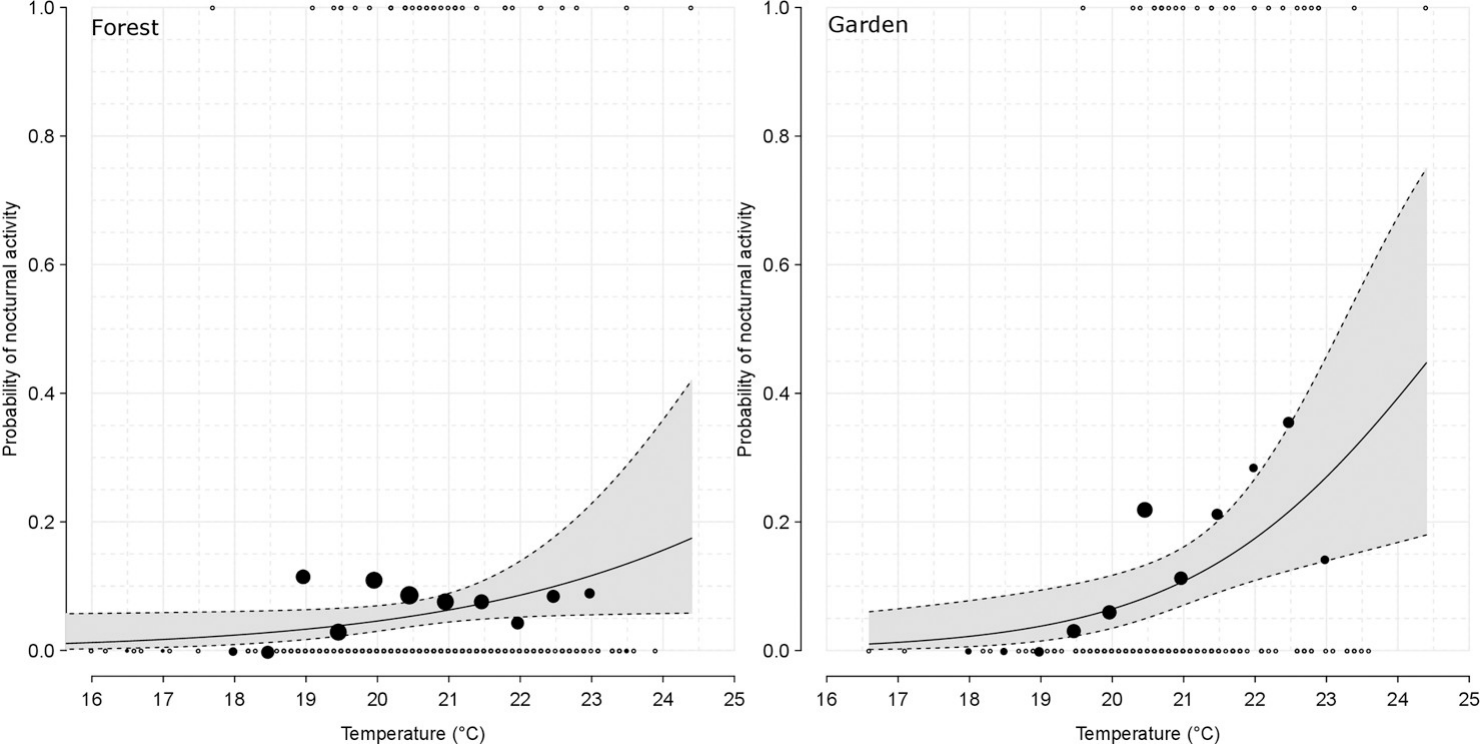
Influence of temperature (°C) on the probability of observing nocturnal activity by camera traps in chimpanzees from Kibale National Park, Uganda according to the habitat (forest or fields). Left is forest and right is field habitat. Points represent the raw data either under binary form (white points) or averaged (black points). The plain black line depicts the response of the model. It depicts the influence of the temperature while averaging the effects of all other variables. Namely, continuous z-transformed variables were set to 0, while categorical predictor habitat type was dummy coded and z-transformed, and finally also set to 0. The gray polygon indicates the 95% confidence interval, borders are depicted by the black dashed lines.

Nocturnal activities both in the forest and fields were more frequent when daytime temperatures were higher (forest estimate±sd: 0.464±0.227, χ^2^ = 4.189, df = 1, *p*-value = 0.041; garden estimate±sd: 0.674±0.235, χ^2^ = 8.769, df = 1, *p*-value = 0.003) (Table 4 and Fig 4). On the other hand, rainfall during the day had no impact on the probability to detect chimpanzees at night (forest: χ^2^ = 2.391, df = 1, *p*-value = 0.122, fields: χ^2^ = 0.478, df = 1, *p*-value = 0.489) (Table 4).

#### Human avoidance or inter-species competition

Moon illumination had no influence on chimpanzee’s nocturnal activity rate in either the forest or the fields (forest: χ^2^ = 0.066, df = 1, *p*-value = 0.798, garden: χ^2^ = 0.109, df = 1, *p*-value = 0.742) (Table 4). Chimpanzees showed nocturnal activities for all possible lunar phases (Fig 2B).

#### Complement dietary requirements hypothesis

**Fig 5:**
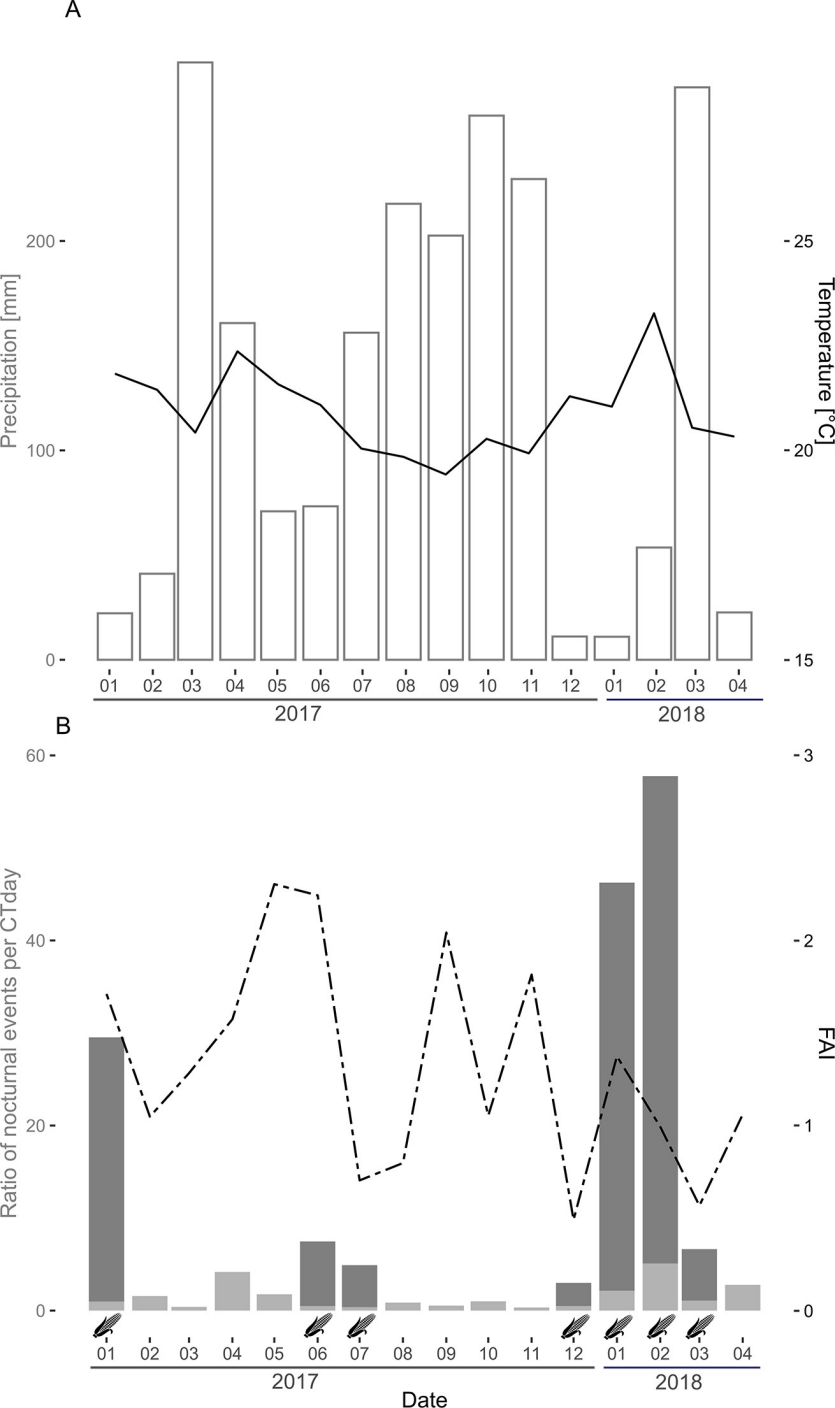
Monthly representation of meteorological factors, food abundance and nocturnal events of chimpanzees according to the location of the camera trap (forest or garden) in Kibale National Park, Uganda. A: Bars represent the precipitation in mm per month. The plain black line depicts the mean temperature per month over the study period. B: Bars represent the monthly ratio obtained with the number of nocturnal events by chimpanzees taking into account the sampling effort with trap days (CTDay) according to their location (dark gray represents gardens and light gray represents forest). The dashed black line depicts the food availability index of ripe fruits (FAI) each month over the study period. The maize pictogram illustrates the monthly presence of maize in fields.

i. The FAI, a proxy for estimating food abundance, varied between 0.49 and 2.30 during the study period (mean±sd = 1.28±0.48). It did not significantly improve our model fit and was not correlated with nighttime events (forest: χ^2^ = 0.116, df = 1, *p*-value = 0.734, fields: χ^2^ = 0.915, df = 1, *p*-value = 0.339; Table 4 and Fig 5).

ii. Data obtained from SCP patrols on maize availability (7 months out of the 15 of the study period) and chimpanzee presence in the fields showed that they visited the fields only during the maize season. However, within the forest the presence of maize did not influence the rate of nocturnal activity (χ^2^ = 1.238, df = 1, *p*-value = 0.266; Table 4).

## Discussion

Our study aimed to document the nocturnal behavior of wild chimpanzees in the context of a forested ecosystem in a protected area surrounded by agriculture. Among the plantations, maize is a highly nutritious and attractive crop, but farmers actively protect their fields from wildlife incursion during daylight hours. We investigated whether this context may explain the high frequency of nocturnal activity observed in a preliminary study in this particular location [14] compared to surveys conducted in 22 other sites [15] where nocturnal activities were less frequent. Our data collection spanned 15 consecutive months, using camera-traps set up within the core area and at the forest-maize fields interface. The data showed that the proportion of nocturnal activity within the forest zone (3.30%) was within the range of that observed in other sites also using camera traps (1.80% the mean of 22 African sites [15]. However, when examining the camera traps set at the forest-field edge, we discovered a very high proportion of nocturnal activity (41.80%), raising the total proportion of nocturnal occurrences within and at the edge of the Sebitoli’s home range to 12.90%, the highest proportion ever recorded in the chimpanzee sites studied (9.58% was the highest frequency in Kayan, Senegal [15]). Our results partially validated some initial predictions: 1) the harsh meteorological conditions reduction strategy where more nocturnal activity in forest and garden occurred after hot days but not after heavy rainfalls, 2) the human avoidance strategy where smaller groups entered the garden at night but nocturnal activity overall was not related to moon illumination but 3) the complement dietary requirements strategy was not confirmed as nocturnal activities in the forest was not correlated to the presence of maize, and nocturnal activities in both locations were not correlated with the wild fruit availability.

Primate sleep patterns are highly variable across species, and might be the result of compromises between necessary good sleep quality and favorable foraging conditions, for instance with reduced predation risk or clement environmental conditions [85, 86]. At night, the main activity happened at the beginning of the night, this activity being feeding on maize crops, a high calorific resource at the edge of the forest [14]. This resource is not easily accessible during the day without risk to life, due to farmers actively protecting their fields. This activity pattern was unmatched in the forest. There, chimpanzees mostly travelled around twilight, presumably to reach their nesting site. For both habitats, we observed that nocturnal behaviors coincided with high day-time temperatures, which has been noted in other chimpanzee communities (Fongoli community in Senegal [24]; 22 communities across Africa [15]) and for other cathemeral species (*Eulemur macao* [7]; *Eulemur rubriventer* and *Eulemur fulvus rufus* [87]; *Eulemur collaris* [88]). However, this propensity for being active at night remained unaffected by rainfall. Although these results closely resemble Taggs et al. [15], they are puzzling since heavy rains inhibits chimpanzee activities during the day as they are reluctant to move on wet ground [89], thus compressing their foraging window. However, the many arboreal supports in this forest can offer protection and the opportunity to move around despite heavy rainfalls. Conversely, the potential stress of low rainfall has been demonstrated in a dry savanna context [90–92] and may also occur in the future for this studied community, but quite unlikely. Indeed, Uganda had experienced a temperature increase of 0.15–0.5°C every decade since 1960 [93, 94], rainfall had decreased by 28% over the last 32 years and the dry season is becoming longer in the Northern part of Kibale National Park [95]. Nonetheless, in this mid-altitude forest crossed by two main rivers, the stress of low rainfall may not be felt by chimpanzees as water sources are present.

Since visibility decreases at night for farmers guarding the fields, primates’ nocturnal activity has also been assumed to be a strategy to reduce the risk associated with human interaction. Because they also rely primarily on vision for foraging (e.g illumination intensity ranges between 1 to 85 lux for feeding activity and full-moon nights offer 0.3 lux [96]), the frequency of such nocturnal activity should not be equal across all nights, but positively correlated with moon illumination [14, 18, 19, 25, 97, 98]. Here, contradicting with our predictions, we did not observe a linear variation in the frequency of nocturnal activity in the forest or in the fields, with moon illumination. In deep forest, it is certain that even a full-moon would not facilitate activities such as feeding, which usually requires substantial illumination to be easily performed [96]. In fields, an open habitat, although chimpanzees might benefit more significantly from higher lunar illumination, the lack of variation in nocturnal activity with moon phases could be due to the fact that the main attraction driver is maize maturity, for which there is no reason to correlate with lunar phases. In addition, our data could be biased by the fact that clouds were not taken into account and could have greatly influenced the actual light transmitted to the ground.

Despite the common misconception that the tropical environment is permanently food rich, there are fluctuations in overall food availability [63]. It can result in variation in calorific intakes for some species unless they diversify their diet over time (e.g. in western lowland gorillas [99]). In the case of chimpanzees, they have generally diversified their diet to the point of incorporating crop foods [35, 40] as they have been exposed to agriculture for a significant amount of time (earliest evidence for cultivation in western Uganda reported at 4,800 BP [100]). Chimpanzees following the optimal foraging strategies can switch from one food source to another to maintain their energy balance, and we therefore expected that chimpanzees would rely more on crops when wild fruits are less available [39, 40, 45, 101]. Yet, as in Tweheyo et al. [102], crop-foraging events occurred when plenty of fruits were available. Indeed, the FAI, the index used to estimate availability of fruits, had no impact on the likelihood of chimpanzees foraging maize. If the quantity argument does not seem that significant, it could ultimately be explained by the fact that maize fields are a reliable, more palatable and nutritive food sources [37, 38] since they contain less non-digestible fibers, more protein [103] and are more energy dense [14] while necessitating less handling overall and no climbing when compared to wild food [104]. No matter the availability of such wild food, feeding from these more nutrient-rich crops would still offset the cost associated with foraging in the fields to a much greater extent than, wild food items [105]. In our study, evidence of chimpanzees in the fields only appeared during maize season. More raids happened during the true night, as opposed to the twilight hours, which is consistent with the estimated reduced vigilance of farmers. In addition, reducing the cost associated with foraging can be mitigated not only by better timing incursions to the crop fields, but also by minimizing the travelled distance to access these areas. Given that chimpanzees favor night field raids, and generally sleep in nests that they build at the end of the day [106], we hypothesize that they would actually nest closer to maize gardens. This should explain why we do not witness an increase of nocturnal activity in the forest during the maize season. The decision to sleep a full night or to wake up for nocturnal activity appeared to be a trade-off [107, 108] and can be altered depending upon changing environment, as the opportunity to feed become advantageous at night by avoiding human threats (confirming [14]).

In Sebitoli, the major risk to chimpanzees comes from spears of farmers that may injure them defending their crops if they see them [104], as they are not involved in active hunting and tracking of prey in the forest. This situation can be associated to a predation risk for the chimpanzees. To minimize this risk, grouped individuals can form larger group making it less likely that any given individual will be preyed upon (dilution effect) [109, 110]. However, in the specific case of these raids, having more individuals equates to a greater chance of being spotted by patrolling people [44]. Here, we did observe that in areas where the risk was highest (e.g gardens, day or night), party size was significantly reduced compared to party size in the forest (5 vs 4 individuals without the lone individuals) despite lone individuals were more frequent in the forest, especially adult males [in accordance with 15]. This may be due to them being more likely to engage in nightly territory patrols [111]. This suggests that small groups of two to three individuals attempted to forage in these crops, particularly males who accounted for 31% of individuals observed in garden whereas they account for less than 18% of community’s individuals and are avoided by adult females in general (21% of individuals in garden vs 48% in the community). It has been suggested that it could be a delayed food-for-sex strategy where they showed prowess and enhance affiliative relationships by sharing the crops with females inside the forest [112]. It has also been shown that males occupy a wider range than females [113]. Further investigation has shown that some females who occupy smaller home range away from the peripherical gardens have lower access to them, and this could be the drivers of such a pattern [Couturier et al, in press]. On the other hand, mutilated individuals and immatures were represented according to their distribution in the community and equally during the day and in both habitats. What we therefore assumed to be an increase in their vulnerability, notably to farmers, did not in fact impact on their pattern of space-use or activity, as has been shown for their activities inside the forest. The risk perception may differ across age and/or sex [114].

In conclusion, human activities influence the behavior, the ecology and the sociology of species in many ways [115]. For example, morphological differences and altered life history traits between wild and more urban populations of certain species have been observed [116, 117]. Here, we provide some evidence that behaviors can be impacted as well, based on the day-night foraging pattern of chimpanzees. Behavioral plasticity may in fact buffer habitat disturbances, and might explain why wild chimpanzee populations persist in anthropized environments despite a slow lifecycle [118]. Nonetheless, if we focus here on how wild chimpanzees derive nutritional benefit from human crops, a short-term view presents living in the vicinity of humans as beneficial, however medium to long term views present the downsides of increased exposure to contamination by pathogens from farmers [119] and their livestock, the risks of injury or death by farmers protecting their crops [105]. In addition, in Sebitoli, in the northern part of Kibale National Park, the use of pesticides in the surrounding crops and tea plantations may also have serious consequences for primate health, with numerous possibly related malformations in baboons (*P*. *anubis*) and chimpanzees [120, 121]. Indeed, 13 pesticides were found in Sebitoli water samples that impact on the hormonal system and are possible endocrine disruptors [120, 122]. Further studies need to investigate the impact on the fertility and development of the Sebitoli community over a longer period. In order to prevent crop feeding by chimpanzees, farmers could strengthen their vigilance during the night not only for elephant intrusions but also for chimpanzees, and/or develop effective 24-hour deterrence methods. In addition, we recommend rethinking land-use near the forest interface to reconcile wildlife conservation and human development by relocating maize fields and growing unpalatable crops at the park’s edge to prevent both economic losses and injuries or kills of endangered species. This survey emphasizes the urgent need to work with local communities on the issue of human-wildlife conflict.

## Supporting information

S1 File Ethogram of the chimpanzee main activity recorded in the clip. (DOCX)

S2 File Raw data of video clips information collected and the chimpanzees identified. (XLSX)

S3 File List of the daily environmental information. (CSV)

S4 File Graphics of the residual diagnostics for the two generalized linear models and the DFBetas results. (DOCX)
