# Supplementary material for: Between forest and croplands: Nocturnal behavior in wild chimpanzees of Sebitoli, Kibale National Park, Uganda
Source: PLoS One. 2022 May 6;17(5):e0268132. doi: 10.1371/journal.pone.0268132 (PMC9075648; doi:10.1371/journal.pone.0268132)
Supplement: S1 File — (DOCX) [file pone.0268132.s001.docx]

SUPPLEMENTARY 1. Ethogram of chimpanzees’ main activity recorded in the clip.

| **Behavior** | **Description** |
| --- | --- |
| Travelling | Locomotion, excluding the below behaviors |
| Sitting | Sit on the floor, some movement of head and/or limbs |
| Standing | Stand on the floor, some movement of head and/or limbs |
| Resting | Flat on floor, some movement of head and/or limbs |
| Grooming | Picking through the fur of self or another individual with hand and/or mouth |
| Feeding | Feeding behavior including searching for/manipulating wild items |
| Cropfeeding | Collecting or feeding on maize comb or stem, moving or sitting while carrying maize in hand and/or mouth |
| Other | Behavior that does not fit above categories (e.g. looking at the camera, drinking water, displaying …) |
