# Supplementary material for: Between forest and croplands: Nocturnal behavior in wild chimpanzees of Sebitoli, Kibale National Park, Uganda
Source: PLoS One. 2022 May 6;17(5):e0268132. doi: 10.1371/journal.pone.0268132 (PMC9075648; doi:10.1371/journal.pone.0268132)

SUPPLEMENTARY 3. Graphics of the residual diagnostics for the two generalized linear models and the DFBetas results

Forest model: PresenceForest ~ Temperature.z + Rainfall.log.z + Moon.illumination.z + FAI.z + Maize, offset= NumberCT.z

| DFBetas | Estimate | Min | Max |
| --- | --- | --- | --- |
| Intercept | -3.08348 | -3.12305 | -3.01184 |
| Temperature.z | 0.46419 | 0.40668 | 0.55010 |
| RainfallLog.z | -0.34002 | -0.38010 | -0.28456 |
| Moon.illumination.z | -0.04649 | -0.08076 | -0.01685 |
| FAI.z | 0.09787 | 0.03472 | 0.15323 |
| Maize | 0.58084 | 0.45126 | 0.68194 |


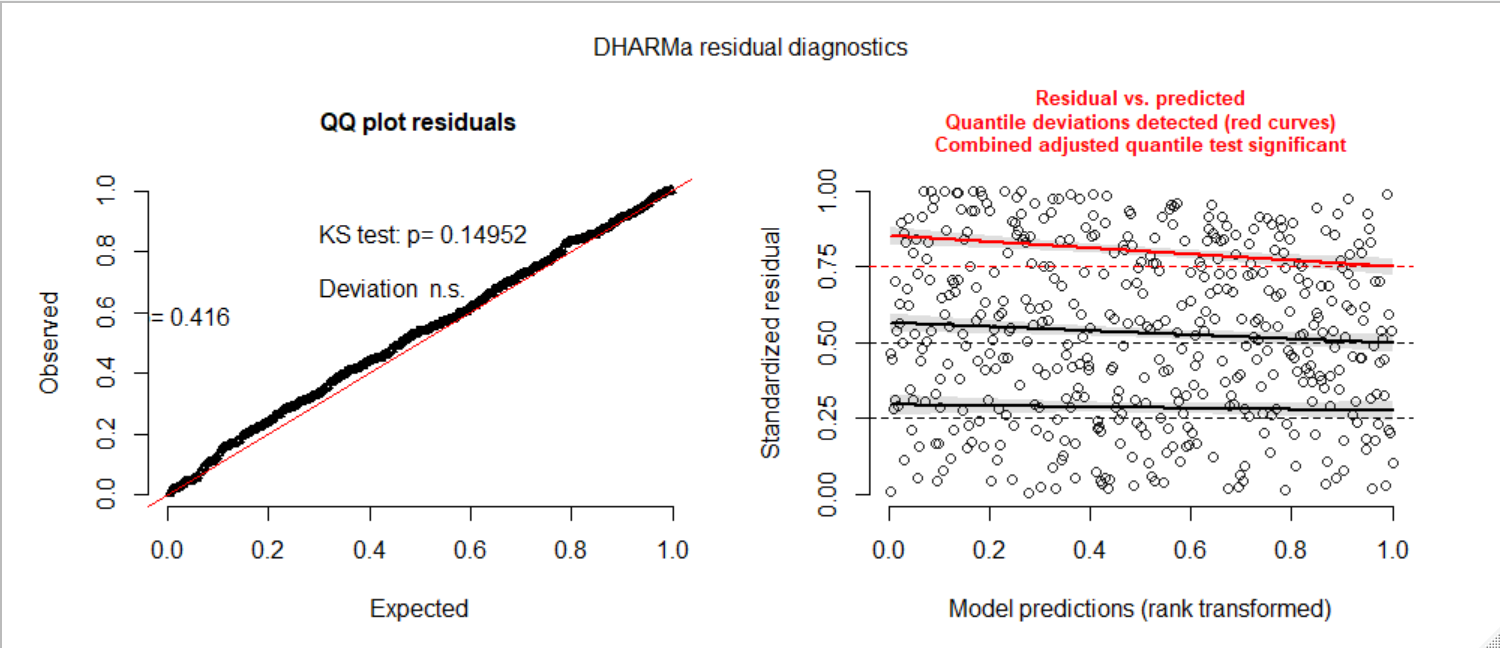


Garden model: PresenceGarden ~ Temperature.z + Rainfall.log.z + Moon.illumination.z + FAI.z, offset= NumberCT.z

| DFBetas | Estimate | Min | Max |
| --- | --- | --- | --- |
| Intercept | -2.21987 | -2.24251 | -2.16700 |
| Temperature.z | 0.67374 | 0.60427 | 0.75689 |
| RainfallLog.z | -0.18931 | -0.23651 | -0.02157 |
| Moon.illumination.z | -0.07688 | -0.12409 | -0.01855 |
| FAI.RF.z | 0.25276 | 0.20187 | 0.29369 |
|  |  |  |  |


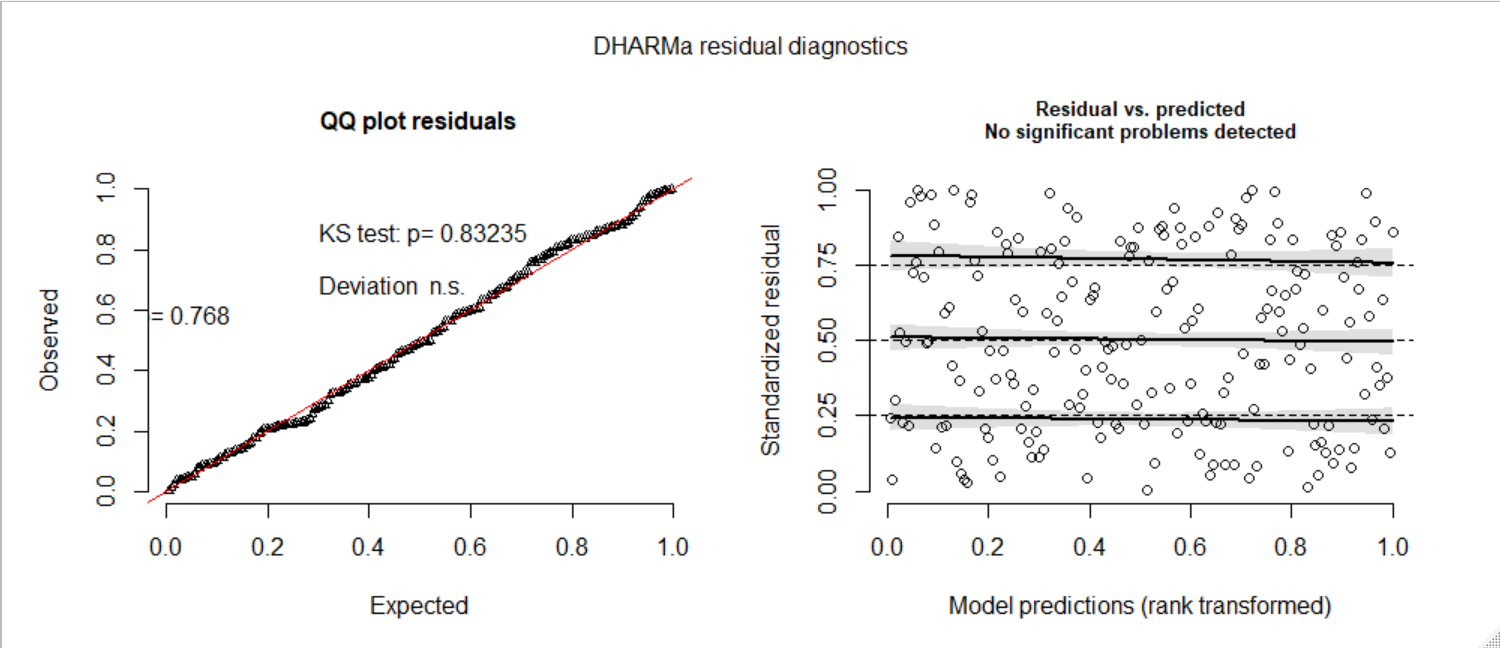

Supplement: S4 File — (DOCX) [file pone.0268132.s004.docx]
